# Supplementary material for: A Comprehensive Assessment of Ultraviolet-Radiation-Induced Mutations in Flammulina filiformis Using Whole-Genome Resequencing
Source: J Fungi (Basel). 2024 Mar 20;10(3):228. doi: 10.3390/jof10030228 (PMC10971301; doi:10.3390/jof10030228)
Supplement: Supplementary file 1 [file jof-10-00228-s001.zip › Supplementary Material S8/KEGG annotation/out/64381550635650.os/KO/out_map/map04961.html]

KEGG PATHWAY: Endocrine and other factor-regulated calcium reabsorption - Reference pathway


|  |  |
| --- | --- |
| **Endocrine and other factor-regulated calcium reabsorption - Reference pathway** |  |

[
Pathway menu
| Organism menu
| Pathway entry
| Show description
| User data mapping
]

Calcium (Ca2+) is essential for numerous physiological functions including intracellular signalling processes, neuronal excitability, muscle contraction and bone formation. Therefore, its homeostasis is finely maintained through the coordination of intestinal absorption, renal reabsorption, and bone resorption. In kidney, the late part of the distal convoluted tubule (DCT) and the connecting tubule (CNT) are the site of active Ca2+ transport and precisely regulate Ca2+ reabsorption. Following Ca2+ entry through TRPV5, Ca2+ bound to calbindin-D28K diffuses to the basolateral side, where it is extruded into the blood compartment through NCX1 and to a lesser extent PMCA1b. In the urinary compartment, both klotho and tissue kallikrein (TK) increase the apical abundance of TRPV5. In the blood compartment, PTH, 1,25(OH)2D3 and estrogen increase the transcription and protein expression of the luminal Ca2+ channels, calbindins, and the extrusion systems.

|  |  |
| --- | --- |
| Reference pathway | 100% |
